# Analytical and Clinical Performance of the NeuMoDx™ Platform for Cytomegalovirus and Epstein–Barr Virus Viral Load Testing

**DOI:** 10.3390/v16050671

**Published:** 2024-04-25

**Authors:** Lindsay Coupland, Katy Woodward, Samir Dervisevic, Rachel Hale, Stephen Brolly

**Affiliations:** Microbiology Department, Eastern Pathology Alliance, Norfolk and Norwich University Hospital NHS Foundation Trust, NRP Innovation Centre, Norwich Research Park, Colney, Norwich NR4 7GJ, UK; katy.woodward@nnuh.nhs.uk (K.W.); samir.dervisevic@nnuh.nhs.uk (S.D.); rachel.hale@nnuh.nhs.uk (R.H.); stephen.brolly@nnuh.nhs.uk (S.B.)

**Keywords:** cytomegalovirus, Epstein–Barr virus, immune deficiency, DNA viral load monitoring, NeuMoDx CMV Quant Assay, NeuMoDx EBV Quant Assay

## Abstract

DNA assays for viral load (VL) monitoring are key tools in the management of immunocompromised patients with cytomegalovirus (CMV) or Epstein–Barr virus (EBV) infection. In this study, the analytical and clinical performances of the NeuMoDx™ CMV and EBV Quant Assays were compared with artus CMV and EBV QS-RGQ Kits in a primary hospital testing laboratory. Patient plasma samples previously tested using artus kits were randomly selected for testing by NeuMoDx assays. The NeuMoDx CMV Quant Assay and artus CMV QS-RGQ Kit limits of detection (LoDs) are 20.0 IU/mL and 69.7 IU/mL, respectively; 33/75 (44.0%) samples had CMV DNA levels above the LoD of both assays. The Pearson correlation coefficient was 0.9503; 20 samples (60.6%) had lower NeuMoDx CMV quantification values versus the artus kit. The LoD of the NeuMoDx EBV Quant Assay and artus EBV QS-RGQ Kit are 200 IU/mL and 22.29 IU/mL, respectively; 16/75 (21.3%) samples had EBV DNA levels above the LoD of both assays. The Pearson correlation coefficient was 0.8990. EBV quantification values with the NeuMoDx assay were higher versus the artus kit in 15 samples (93.8%). In conclusion, NeuMoDx CMV and EBV Quant Assays are sensitive and accurate tools for CMV and EBV DNA VL quantification.

## 1. Introduction

The ubiquitous human pathogens, cytomegalovirus (CMV) and Epstein–Barr virus (EBV) are common infections globally, with an estimated prevalence of over 90% in some populations [1,2]. Like all herpesviruses, CMV and EBV are highly host-adapted, capable of establishing latency and persisting for the life of the individual; however, during periods of immunosuppression, they can reactivate and switch to the lytic state, progressing to disseminated infection [1,2,3]. Infections with CMV and EBV are generally asymptomatic [2,4], but can cause serious disease complications in patients with immunodeficiency [4,5]: a high CMV viral load (VL) is often observed prior to end-organ disease following solid organ or stem cell transplantation [2,6], and a high EBV VL has been linked to lymphoproliferative disease [1] or to the onset of non-Hodgkin’s lymphomas in patients with human immunodeficiency virus [1].

DNA assays for VL monitoring are key tools in informing the clinical management of immunocompromised patients with a CMV or EBV infection [6,7,8]. Available semi- and fully automated platforms include the *m*2000 RealTi*m*e System (Abbott Molecular Inc., Des Plaines, IL, USA), QIAsymphony® RGQ system (QIAGEN GmbH, Hilden, Germany), eMAG^®^/eSTREAM^®^ (bioMérieux, Inc., Marcy-l’Etoile, France), Panther^®^ System (Hologic Inc., Marlborough, MA, USA), Cobas^®^ 4800/5800/6800/8800 Systems (F. Hoffmann La-Roche Ltd., Basel, Switzerland), and NeuMoDx™ 96 and 288 Molecular Systems (QIAGEN GmbH, Hilden, Germany) [9,10,11,12,13,14]. A goal of laboratory medicine is that patient sample results are comparable, independently of the laboratories or methods used [15], to enable accurate data interpretation and development of common cut-offs for therapeutic decision making [16].

International standards (i.e., such as those produced by the World Health Organization [WHO] for quantitative detection of CMV and EBV) [16,17] and reporting in international units (IU)/mL (rather than DNA copies/mL) help to standardise the results across assays [15]. However, variability is still observed when testing clinical samples in the laboratory, particularly between institutions [9,16,17]; therefore, validation procedures are required for each new assay introduced [18].

## 2. Patients and Methods

### 2.1. Objectives

This study compared the analytical and clinical performance of the NeuMoDx CMV Quant Assay and the NeuMoDx EBV Quant Assay with established reference methods, the artus CMV QS-RGQ Kit and the artus EBV QS-RGQ Kit, respectively, in a primary hospital testing laboratory.

### 2.2. Study Design

#### 2.2.1. Samples

Human plasma samples were collected from patients with haematological diseases requiring follow-up testing for CMV and EBV infection between 2018 and 2020.

#### 2.2.2. Assays

The artus CMV and EBV QS-RGQ Kits constitute ready-to-use systems for the detection of CMV and EBV DNA using a polymerase chain reaction on the Rotor-Gene Q Instrument (QIAGEN GmbH, Hilden, Germany). The NeuMoDx CMV and EBV Quant Assays, performed on the NeuMoDx™ 96 or 288 Molecular Systems (NeuMoDx™ Molecular, a QIAGEN company, Ann Arbor, MI, USA), are automated, in vitro, diagnostic nucleic acid amplification tests designed for the quantitation of CMV and EBV DNA, respectively, in fresh and frozen human plasma samples [10,11].

All assays were performed according to the manufacturer’s instructions [10,11,19,20]. Procedural methods for the assays are provided in the Supplementary Materials.

Table 1 shows the manufacturer-reported analytical sensitivity and assay characteristics of the NeuMoDx CMV Quant Assay. The limits of detection (LoDs) of the NeuMoDx CMV Quant Assay and the artus CMV QS-RGQ Kit are 20.0 IU/mL and 69.7 IU/mL, respectively, as determined by the assay manufacturers and using the provided conversion factor to adapt copies/mL to IU/mL [10,21]. 

### 2.3. Determining Analytical Sensitivity

The analytical sensitivity of the NeuMoDx CMV and EBV Quant Assays was previously determined by the manufacturer [10,11]. Confirmation of the analytical sensitivity of the NeuMoDx CMV and EBV Quant Assays was carried out using the Qnostics CMV Analytical Q Panel 02 (CMVAQP02-B; Qnostics, Glasgow, UK) and the Qnostics EBV Analytical Q Panel 03 (EBVAQP03-B), respectively.

### 2.4. Determining Clinical Performance

Surplus ethylenediaminetetraacetic acid (EDTA)-treated human plasma samples, previously tested using the artus CMV and EBV QS-RGQ Kits, were randomly selected for testing by the NeuMoDx CMV and EBV Quant Assays. The samples were stored at −20 °C or lower until testing; all samples underwent a single freeze–thaw cycle before processing.

### 2.5. Data Analyses

Concordance between the assay VL detections was assessed quantitatively. Pearson’s correlation coefficient and Deming regression were calculated for all samples with quantitative results from both assays to measure the overall correlation between the NeuMoDx and artus assays. A Bland–Altman plot was created to analyse assay agreement, by measuring the mean differences between log_10_ IU/mL values reported by the NeuMoDx and artus assays versus the average log_10_ IU/mL generated by each assay for each sample. The estimated agreement interval was defined as ±1.96 standard deviations (SD) from the overall mean difference (the threshold within which 95% of the observations lie within a normally distributed dataset) [22]. Analyses were conducted using Microsoft Excel 2010 and MedCalc Software Version 19.3.1.

**Table 1:** Manufacturer-reported analytical sensitivity and assay characteristics.

	Assay	Plasma Input Volume (µL)	Target Gene(s)	LoD (IU/mL)	LLoQ (IU/mL)	ULoQ (IU/mL)
CMV	NeuMoDx CMV Quant Assay ^a^	550 [10]	*UL54* and *UL71* [23]	For variant gB1: 17.5 Across genotypes: 20.0 [10]	20.0 [10]	8.0 log_10_ [10]
	artus CMV QS-RGQ Kit ^b^	1200 [24]	*MIE* [20]	69.7 [21] (converted from 42.5 copies/mL)	1.30 × 10^2^ [21] (converted from 79.4 copies/mL)	1.64 × 10^8^ [21] (converted from 1.00 × 10^8^ copies/mL)
EBV	NeuMoDx EBV Quant Assay ^c^	250 [11]	*BALF5* and *BXFL1* [23]	200 [11]	200 [11]	8.0 log_10_ [11]
	artus EBV QS-RGQ Kit ^b^	1200 [25]	*EBNA-1* [26]	22.29 [27] (converted from 157 copies/mL)	8.96 × 10^1^ [27] (converted from 631 copies/mL)	1.42 × 10^6^ [27] (converted from 1.00 × 10^7^ copies/mL)

^a^ Traceable to the 1st WHO International Standard for CMV [10]. ^b^ artus CMV and EBV assays were developed prior to the availability of the WHO international standards. Performance characteristics are, therefore, given in copies/mL, and a conversion factor is available for customers who prefer to report in IU/mL, traceable to the 1st WHO International Standard for CMV and EBV. EBV: 1 copy/mL corresponds to 0.142 IU/mL for detection of EBV DNA derived from EDTA-treated human plasma on the Rotor-Gene Q. CMV: 1 copy/mL corresponds to 1.64 IU/mL for detection of CMV DNA derived from EDTA-treated human plasma on the Rotor Gene Q. ^c^ Traceable to the 1st WHO International Standard for EBV [11]. CMV, cytomegalovirus; EBV, Epstein–Barr virus; EDTA, ethylenediaminetetraacetic acid; IU, international units; LLoQ, lower limit of quantitation; LoD, limit of detection; ULoQ, upper limit of quantitation; WHO, World Health Organization.

## 3. Results

### 3.1. CMV Assay Comparison

#### 3.1.1. Analytical Sensitivity and Performance Characteristics of the NeuMoDx CMV Quant Assay

The NeuMoDx CMV Quant Assay detected and quantified CMV DNA in all samples of the Qnostics CMV panel, including the sample with the lowest target concentration of 30 IU/mL (Supplementary Table S1), in agreement with the manufacturer-reported LoD and lower limit of quantitation (LLoQ). The bias between panel target concentration and the value quantified by the NeuMoDx CMV Quant Assay was <0.5 log_10_ IU/mL for all panel samples with a quantitative value.

#### 3.1.2. Clinical Performance of the NeuMoDx CMV Quant Assay

Overall, 75 EDTA-treated human plasma samples were processed using the NeuMoDx CMV Quant Assay (Supplementary Table S2). Table 2 shows a comparison of the CMV viral loads attained using the NeuMoDx CMV Quant Assay and the artus CMV QS-RGQ Kit. Thirty-three (44.0%) samples had CMV DNA levels detectable above the LoD of both assays, 14 (18.7%) samples had CMV DNA levels undetected by both assays, and the remaining 28 (36.0%) samples had CMV DNA levels outside of the quantification limits of at least one assay. Supplementary Table S3 provides a comparison of the CMV DNA levels between the assays for all 75 samples. 

#### 3.1.3. Correlation between the NeuMoDx CMV Quant Assay and the Artus CMV QS-RGQ Kit

**Figure 1:**
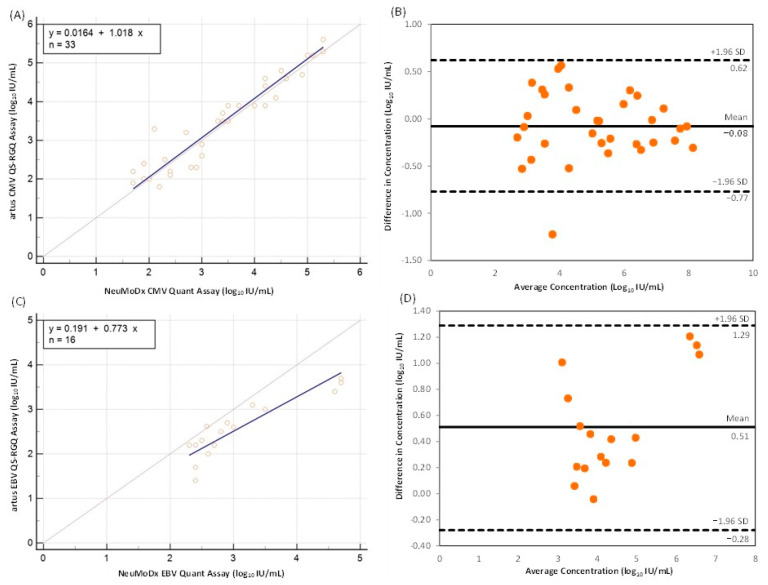
Deming linear regression and Bland–Altman plot for NeuMoDx versus artus for CMV assays ((**A**,**B**), respectively) and EBV assays ((**C**,**D**), respectively). CMV, cytomegalovirus; EBV, Epstein–Barr virus; IU, international units; SD, standard deviation.

Thirty-three CMV DNA-positive samples with DNA levels above the LoD of both tests were used to generate the linear regression model. Deming regression was performed for the samples with quantified results by the NeuMoDx CMV Quant Assay and artus CMV QS-RGQ Kit (Figure 1A). The Pearson correlation coefficient was 0.9503 (95% confidence interval [Cl]: 0.9008–0.9754), indicating a strong correlation.

The Bland–Altman plot showed the difference between log_10_ IU/mL values by the NeuMoDx CMV Quant Assay and artus CMV QS-RGQ Kit versus the average log_10_ IU/mL generated by both assays for each sample (Figure 1B). The mean bias (±1.96 SD) between the NeuMoDx CMV Quant Assay and the artus CMV QS-RGQ Kit was −0.08 (±0.36) log_10_ IU/mL; the limits of agreement were −0.77 and 0.62 log_10_ IU/mL. A discrepancy in the quantification value between the NeuMoDx CMV Quant Assay and the artus CMV QS-RGQ Kit was detected for one sample that fell outside the estimated agreement interval.

The CMV DNA quantification values reported by the NeuMoDx CMV Quant Assay were lower than for the artus CMV QS-RGQ Kit in 20 samples (60.6%; Supplementary Table S3).

### 3.2. EBV Assay Comparison

#### 3.2.1. Analytical Sensitivity and Performance Characteristics of the NeuMoDx EBV Quant Assay

Table 1 shows manufacturer-reported analytical sensitivity and assay characteristics of the NeuMoDx EBV Quant Assay. The LoD of the NeuMoDx EBV Quant Assay and the artus EBV QS-RGQ Kit are 200 IU/mL and 22.29 IU/mL [11,27]. 

The NeuMoDx EBV Quant Assay detected EBV DNA in all samples of the Qnostics EBV panel (Supplementary Table S4). A quantitative result was obtained for all panel samples, excluding the sample with the lowest target concentration (below the assay LoD of 200 IU/mL), which was detected but not quantifiable. The bias between panel target concentration and the value quantified by the NeuMoDx EBV Quant Assay was <0.5 log_10_ IU/mL for all panel samples with a quantitative value.

#### 3.2.2. Clinical Performance of the NeuMoDx EBV Quant Assay

The same 75 EDTA-treated human plasma samples that were tested for CMV were processed using the NeuMoDx EBV Quant Assay. Table 2 shows a comparison of the EBV DNA levels attained using the NeuMoDx EBV Quant Assay and the artus EBV QS-RGQ Kit. Sixteen (21.3%) samples had EBV DNA levels falling within the quantification limits of both assays, 38 (50.7%) samples had EBV DNA levels undetected by both assays, and the remaining 21 (28.0%) samples had EBV DNA levels outside of the quantification limits of at least one assay. Supplementary Table S5 provides a comparison of EBV DNA levels between the assays for all 75 samples. 

#### 3.2.3. Correlation between the NeuMoDx EBV Quant Assay and the Artus EBV QS-RGQ Kit

Sixteen EBV DNA-positive samples within the linear range of both tests were used to generate the linear regression model. Deming regression was performed for samples with quantified results and the Pearson correlation coefficient was 0.8990 (95% Cl: 0.7276–0.9648), indicating a strong correlation (Figure 1C).

The Bland–Altman plot showed the difference between log_10_ IU/mL values on the NeuMoDx EBV Quant Assay and the artus EBV QS-RGQ Kit versus the average log_10_ IU/mL generated by both assays for each sample (Figure 1D). The mean bias (±1.96 SD) between the NeuMoDx EBV Quant Assay and the artus EBV QS-RGQ Kit was 0.51 (±0.40) log_10_ IU/mL; the limits of agreement were −0.28 and 1.29 log_10_ IU/mL. No discrepancies in quantification values between the NeuMoDx EBV Quant Assay and the artus EBV QS-RGQ Kit were detected.

The EBV quantification values reported by the NeuMoDx EBV Quant Assay were higher than for the artus EBV QS-RGQ Kit in 15 samples (93.8%; Supplementary Table S5).

## 4. Discussion

This validation study confirmed the manufacturer-reported analytical sensitivity for the NeuMoDx CMV and EBV Quant Assays using external quality assessment panels, with accurate quantification of VL compared with the equivalent artus reference methods.

The reliability of assay comparisons depends upon the accuracy of the reference method (i.e., artus QS-RGQ CMV and EBV Kits) in reporting the results. However, the higher proportion of positive results with the NeuMoDx CMV Quant Assay versus the artus CMV QS-RGQ Kit may actually represent an improvement in the detection of CMV DNA at lower target levels compared to the artus CMV QS-RGQ Kit, as reflected in the NeuMoDx assay’s lower LoD and LLoQ. To illustrate, a high proportion of samples (*n* = 20; 26.7%) were quantifiable by the NeuMoDx CMV Quant Assay, but undetected or below the LoD by the artus kit; however, there were no such samples with the EBV assays. In contrast, 12 (16.0%) samples were undetectable by the NeuMoDx EBV Quant Assay, but detectable by the artus kits (albeit below or close to the LoD), while there were only six (8.0%) such samples for the CMV assay; these findings reflect the higher LoD of the NeuMoDx EBV Quant Assay versus the artus kit. This difference may also be related to the different targets of the NeuMoDx EBV Quant Assay (*BALF5* and *BXFL1*), compared to the artus EBV kit (*EBNA-1*), as the assay sensitivity may vary depending on the specific gene segment targeted [28].

These findings are supported by Mourik et al., in which 35/450 (7.8%) samples had CMV DNA undetected by the m2000 or an established laboratory-developed test (LDT), but were quantifiable using the NeuMoDx CMV Quant Assay [23]; conversely, in 15 samples (3.3%), the CMV DNA was detectable using the m2000 or LDT, but not using the NeuMoDx CMV Quant Assay. Similarly to the present results, the EBV DNA was undetected by the m2000 or LDT, but quantifiable using the NeuMoDx EBV Quant Assay in 8/432 samples (1.9%), and the EBV DNA was detectable using the m2000 or LDT, but not using the NeuMoDx EBV Quant Assay in 43/432 samples (10.0%). Herdina et al. also identified divergent quantifiable results in 25/279 (9.0%) samples and 19/245 (7.8%) samples using the NeuMoDx CMV Quant Assay and NeuMoDx EBV Quant Assay, respectively, that had previously quantifiable virus by reference tests [29].

Therefore, the NeuMoDx CMV Quant Assay may detect increasing CMV VLs earlier than other assays. Luciani et al. suggested that, for CMV DNA monitoring in transplant recipients, during the first two weeks of treatment, the NeuMoDx CMV Quant Assay tended to estimate higher VLs in plasma samples compared to R-GENE^®^ (bioMérieux, Inc., Marcy-l’Etoile, France). However, in whole blood samples, this was not noticeable after 2 weeks of treatment. While that study is not directly comparable to the present study, as it pertained to different sample inputs (plasma versus whole blood), the authors concluded that use of the NeuMoDx assay would not result in differences in the clinical treatment of CMV infections in transplanted patients [30]. However, it was noted that the random-access properties of the NeuMoDx platform could offer significant advantages versus the R-GENE in a hospital setting, such as a shorter time to result (<4 h versus 12–24 h with the R-GENE), allowing medical staff to receive the result the same day (during duty hours) [30].

The NeuMoDx assays offer operational benefits versus the artus kits, including minimal hands-on time, a single pipetting step per sample to reduce errors, and all steps of the NeuMoDx 96 Molecular System diagnostic process being fully automated, including data analysis and interpretation of results (compared to the need for operator transfers on the thermocycler for the artus QS-RGQ CMV and EBV Kits). Sample-to-result turnaround time is also shorter (approximately 60–80 min versus 3.5 h for the artus kits). This is particularly important when managing patients with iatrogenic or acquired immunosuppression, or in paediatric settings. The possibility for emergency prioritisation of samples (‘STAT’ functionality) of the NeuMoDx platforms also allows for urgent samples to be tested individually, rather than in batches; an advantage for hospital diagnostics [31].

Despite adopting international standards and reporting using IU/mL, meaningful inter-assay result comparisons for VL testing of CMV and EBV remain limited by inter-laboratory discordance [16,32,33]. This disagreement, and the poor commutability of standards, although multifaceted, may relate to the amplicon size and fragmentation of circulating viral DNA [32,33]. Using fragmented reference material may improve commutability and inter-assay agreement, particularly for CMV [32]. Defining and mitigating the causes of discordance is required to further advance the clinical utility of CMV and EBV assays [32].

Interpretation of our study findings may be limited by the sample storage and freeze–thaw step prior to testing on the NeuMoDx platforms. This may have led to the potential degradation of viral material, possibly contributing to the high proportion of observed samples undetectable by the NeuMoDx EBV Quant Assay, but detectable by the artus EBV QS-RGQ assay. Herdina et al. and Mourik et al. also include storage conditions and the use of archived clinical samples as potential influencing factors on performance outcomes. Unfortunately, insufficient archived clinical samples remained to perform additional analyses to compare the results with the respective reference tests [23,29].

Other influencing factors include the potential impact of fragmentation and amplicon size on quantification [32], and the lower input volume of plasma and higher LoD of the NeuMoDx EBV Quant Assay compared to the artus kit. Lower CMV quantification values were observed in 60.6% of samples by the NeuMoDx CMV Quant Assay, compared to the artus CMV QS-RGQ Kit, despite the mean bias being only −0.08.

A new iteration of the NeuMoDx EBV Quant Assay (NeuMoDx EBV Quant 2.0) with an improved LoD [34] is now available, which should offer improvements in sensitivity.

## 5. Conclusions

In conclusion, the NeuMoDx CMV and EBV Quant Assays provide highly sensitive and accurate results for CMV and EBV DNA quantification compared to the artus QS-RGQ Kits, with operational advantages. However, the LoD of the NeuMoDx EBV Quant Assay is higher than for the artus EBV QS-RGQ Kit, and some low-level EBV samples were not detected; the clinical significance of this is unclear, and warrants investigation.

## Figures and Tables

**Table 2:** Quantitative comparison of CMV and EBV viral loads for the NeuMoDx CMV and EBV Quant Assays compared with the artus CMV and EBV QS-RGQ Kit.

		Artus CMV QS-RGQ Kit			
		≥69.7 IU/mL ^a^	<69.7 IU/mL ^a^	Not Detected	Total
NeuMoDx CMV Quant Assay	≥20.0 IU/mL ^b^	33	15 ^c^	5 ^d^	53
	<20.0 IU/mL ^b^	0	1	1	2
	Not detected	0	6	14	20
	Total	33	22	20	75
		Artus EBV QS-RGQ Kit			
		≥22.29 IU/mL ^a^	<22.29 IU/mL ^a^	Not detected	Total
NeuMoDx EBV Quant Assay	≥200 IU/mL ^b^	16	0	0	16
	<200 IU/mL ^b^	2 ^e^	6	1	9
	Not detected	0	12	38	50
	Total	18	18	39	75

^a^ Manufacturer-stated LoD for the artus CMV and EBV QS-RGQ Kits. ^b^ Manufacturer-stated LoD for the NeuMoDx CMV and EBV Quant Assays. ^c^ NeuMoDx CMV Quant Assay results ranged from 22–190 IU/mL. ^d^ NeuMoDx CMV Quant Assay results ranged from 25–76 IU/mL. ^e^ artus EBV QS-RGQ Kit results ranged from 33.2–39.5 IU/mL. CMV, cytomegalovirus; EBV, Epstein–Barr virus; IU, international units; LoD, limit of detection.

## Data Availability

The data presented in this study are available on request from the corresponding author (Lindsay Coupland).

## Supplementary Materials

The following supporting information can be downloaded at: https://www.mdpi.com/article/10.3390/v16050671/s1, Table S1: Analytical sensitivity of the NeuMoDx CMV Quant Assay determined using the Qnostics CMV Analytical Q Panel 02 (CMVAQP02-B).Table S2: Summary of samples used to assess clinical performance of the NeuMoDx CMV and EBV Quant Assays. Table S3: Comparison of the CMV DNA levels attained using the artus CMV QS-RGQ Kit and the NeuMoDx CMV Quant Assay. Table S4: Analytical sensitivity of the NeuMoDx EBV Quant Assay determined using the Qnostics EBV Analytical Q Panel 03 (EBVAQP03-B. Table S5: Comparison of the EBV DNA levels attained using the NeuMoDx EBV Quant Assay and the artus EBV QS-RGQ Kit.
