# Supplementary material for: Analytical and Clinical Performance of the NeuMoDx™ Platform for Cytomegalovirus and Epstein–Barr Virus Viral Load Testing
Source: Viruses. 2024 Apr 25;16(5):671. doi: 10.3390/v16050671 (PMC11125657; doi:10.3390/v16050671)
Supplement: Supplementary file 1 [file viruses-16-00671-s001.zip › viruses-2896427-supplementary.pdf]

Supplementary material

NeuMoDx CMV and EBV Quant Assays

Ethylenediaminetetraacetic acid (EDTA)-treated human plasma samples were tested on the NeuMoDx System by employing automated DNA extraction and real-time polymerase chain reaction amplification, result processing and interpretation. The NeuMoDx CMV Quant Assay and the NeuMoDx EBV Quant Assay on the NeuMoDx 96 Molecular System utilise the NeuMoDx CMV/EBV Quant Test Strips, NeuMoDx CMV/EBV Calibrators, NeuMoDx CMV/EBV External Controls, NeuMoDx Lysis Buffer 1 (for cytomegalovirus [CMV]) and 5 (for Epstein-Barr virus [EBV]) and NeuMoDx general use reagents [10,11]. The NeuMoDx CMV Quant Assay allows the amplification of the *UL54* and *UL71* genes in the CMV genome, and the NeuMoDx EBV Quant Assay targets *BALF5* and *BXFL1* in the EBV genome [23]. The minimum sample volume for CMV and EBV, respectively, was 550 μL and 250 μL of EDTA-treated human plasma (plus excess volume requirements to make it up to 1.2 mL).

artus CMV and EBV QS-RGQ Kits

Purification of DNA was performed from 1.2 mL (including excess volume) of EDTA-treated human plasma using the QIAsymphony DSP Virus/Pathogen Midi Kit on the QIAsymphony SP instrument. The eluates from the sample preparation procedure (initial volume 90 μL; preselected protocol volume 60 μL) containing purified DNA were transferred to the QIAsymphony AS module using the 'integrated run' setup. The assays were transferred to the Rotor-Gene Q real-time polymerase chain reaction (PCR) instrument for PCR.

The CMV RG Master Mix and the EBV RG Master Mix contained the reagents and enzymes for the specific amplification of a 105 bp region of the *MIE* gene in the CMV genome and a 97 bp region of the *EBNA-1* gene in the EBV genome, respectively, allowing the direct detection of the specific amplicons [20, 26]. Both assays contained a second heterologous amplification system to identify possible PCR inhibition. Quantitation standards (CMV QS 1–4 and EBV RG QS 1–4) were included per PCR run to generate a standard curve and allow determination of DNA VL in copies/mL; a negative control (PCR grade water) was also included per PCR run. The results in copies/mL were converted to IU/mL prior to data analysis.

Assay calculations

The quantitation standards are defined as copies/μL in the eluate. The following equation was applied to convert the DNA values determined using the standard curve into copies/mL of sample material.

Result (copies/mL) = Result (copies/μL) × initial elution volume (90 μL)

Sample volume (1 mL)

To convert copies/mL to IU/mL prior to data analysis, the conversion factor for the artus CMV QS-RGQ Kits is as follows: 1 copy/mL corresponds to 1.64 IU/mL for detection of CMV DNA and to 0.142 IU/mL for detection of EBV DNA derived from EDTA-treated human plasma on the Rotor-Gene Q.

Table S1. Analytical sensitivity of the NeuMoDx CMV Quant Assay determined using the Qnostics CMV Analytical Q Panel 02 (CMVAQP02-B).

Sample code	Panel target concentration		NeuMoDx CMV Quant Assay results		Difference in concentration[a]
	log_{10} IU/mL	IU/mL	log_{10} IU/mL	IU/mL	
**CMVAQP02-S01**	5.0	100,000	4.63	43,000	–0.37
CMVAQP02-S02	4.5	30,000	4.11	13,000	–0.39
CMVAQP02-S03	4.0	10,000	3.77	5,900	–0.23
CMVAQP02-S04	3.5	3,000	3.26	1,800	–0.24
CMVAQP02-S05	3.0	1,000	2.64	440	–0.36
CMVAQP02-S06	2.5	300	2.04	110	–0.46
CMVAQP02-S07	2.0	100	1.98	96	–0.02
CMVAQP02-S08	1.5	30	1.30	20	–0.20
CMVAQP02-S09	Not detected	Not detected	Not detected	Not detected	0

[a] NeuMoDx CMV Quant Assay (log_{10} IU/mL) minus panel target concentration (log_{10} IU/mL). CMV, cytomegalovirus; IU, international units.

Table S2. Summary of samples used to assess clinical performance of the NeuMoDx CMV and EBV Quant Assays.

	Tested for CMV and EBV DNA
**Samples collected (number of patients)**	89 (12)
**Samples excluded**	14[a]
**Samples with valid results**	75
**Sample type**	Plasma (frozen)
**Sample collection period**	2018–2020
**Reference method**	artus CMV/EBV QS-RGQ kit
**Test method**	NeuMoDx CMV/EBV Quant Assay

[a] Excluded due to insufficient sample, n = 11; excluded due to indeterminate results with both NeuMoDx CMV and EBV Quant Assays, n = 3. CMV, cytomegalovirus; EBV, Epstein-Barr virus.

Table S3. Comparison of the CMV DNA levels attained using the artus CMV QS-RGQ Kit and the NeuMoDx CMV Quant Assay.

Sample No.	artus CMV QS-RGQ Kit		NeuMoDx CMV Quant Assay	
	Result	Concentration, IU/mL	Result	Concentration, IU/mL
		Detected above the LoD of both assays, n = 33		
2	Detected	195.2	Detected	660
7	Detected	96.8	Detected	79
8	Detected	161,471.1	Detected	95,000
9	Detected	7,683.4	Detected	11,000
10	Detected	149.2	Detected	270
14	Detected	5,061.0	Detected	2,800
16	Detected	2,171.4	Detected	130
17	Detected	8,118.0	Detected	3,500
18	Detected	2,860.2	Detected	2,000
22	Detected	329.6	Detected	180
23	Detected	887.2	Detected	1,100
25	Detected	27,809.5	Detected	15,000
26	Detected	3,166.8	Detected	3,000
29	Detected	425,306.1	Detected	21,0000
30	Detected	165,433.4	Detected	13,0000
31	Detected	55,356.6	Detected	71,000
33	Detected	57,173.7	Detected	32,000
34	Detected	36,265.3	Detected	17,000
35	Detected	2,932.3	Detected	2,800
36	Detected	93.5	Detected	100
37	Detected	236.2	Detected	87
38	Detected	7,304.6	Detected	4,500
39	Detected	39,164.8	Detected	38,000
40	Detected	8,477.2	Detected	17,000
41	Detected	201.7	Detected	740
45	Detected	85.3	Detected	54
66	Detected	216,680.1	Detected	180,000

68	Detected	13,080.6	Detected	23,000
69	Detected	434.6	Detected	930
70	Detected	70.5	Detected	170
74	Detected	123.0	Detected	250
87	Detected	1,600.6	Detected	480
89	Detected	172.2	Detected	51
Detected below the artus LoD but detected above the NeuMoDx LoD, n = 15				
3	Detected	<69.7	Detected	35
4	Detected	<69.7	Detected	51
5	Detected	<69.7	Detected	22
11	Detected	<69.7	Detected	130
12	Detected	<69.7	Detected	98
19	Detected	<69.7	Detected	110
20	Detected	<69.7	Detected	190
24	Detected	<69.7	Detected	58
27	Detected	<69.7	Detected	46
46	Detected	<69.7	Detected	35
71	Detected	<69.7	Detected	60
72	Detected	<69.7	Detected	40
75	Detected	<69.7	Detected	180
77	Detected	<69.7	Detected	72
82	Detected	<69.7	Detected	32
Not detected by artus but detected above the NeuMoDx LoD, n = 5				
6	Not detected	N/A	Detected	59
21	Not detected	N/A	Detected	25
42	Not detected	N/A	Detected	28
73	Not detected	N/A	Detected	50
76	Not detected	N/A	Detected	76
Detected below the artus LoD and detected below the NeuMoDx LoD, n = 1				
81	Detected	<69.7	Detected	<20
Not detected by artus but detected below the NeuMoDx LoD, n=1				
53	Not detected	N/A	Detected	<20
Detected below the artus LoD but not detected by NeuMoDx, n = 6				
44	Detected	<69.7	Not detected	N/A
51	Detected	<69.7	Not detected	N/A
52	Detected	<69.7	Not detected	N/A
80	Detected	<69.7	Not detected	N/A
83	Detected	<69.7	Not detected	N/A
84	Detected	<69.7	Not detected	N/A
Not detected by artus and not detected by NeuMoDx, n = 14				
32	Not detected	N/A	Not detected	N/A
43	Not detected	N/A	Not detected	N/A
49	Not detected	N/A	Not detected	N/A
50	Not detected	N/A	Not detected	N/A
54	Not detected	N/A	Not detected	N/A
55	Not detected	N/A	Not detected	N/A
56	Not detected	N/A	Not detected	N/A
57	Not detected	N/A	Not detected	N/A
58	Not detected	N/A	Not detected	N/A
60	Not detected	N/A	Not detected	N/A
61	Not detected	N/A	Not detected	N/A
62	Not detected	N/A	Not detected	N/A
64	Not detected	N/A	Not detected	N/A
65	Not detected	N/A	Not detected	N/A

CMV, cytomegalovirus; IU, international units; LoD, limit of detection; N/A, not applicable.

Table S4. Analytical sensitivity of the NeuMoDx EBV Quant Assay determined using the Qnostics EBV Analytical Q Panel 03 (EBVAQP03-B).

**Sample code**	**Panel target concentration**		**NeuMoDx EBV Quant Assay results**		**Difference in concentration[a]**
	**log_{10} IU/mL**	**IU/mL**	**log_{10} IU/mL**	**IU/mL**	
EBVAQP03-S01	5.9	800,000	6	950,000	0.1
EBVAQP03-S02	5.6	400,000	5.7	520,000	0.1

EBVAQP03-S03	4.9	80,000	5	91,000	0.1
EBVAQP03-S04	4.6	40,000	4.7	49,000	0.1
EBVAQP03-S05	3.9	8,000	4.1	12,000	0.2
EBVAQP03-S06	3.6	4,000	3.9	7,900	0.3
EBVAQP03-S07	2.9	800	3.3	1,900	0.4
EBVAQP03-S08	2.6	400	2.7	450	0.1
EBVAQP03-S09	1.9	80	<2.3	<200	≥0.4
EBVAQP03-S10	Not detected	Not detected	Not detected	Not detected	0

[a] NeuMoDx EBV Quant Assay (log_{10} IU/mL) minus panel target concentration (log_{10} IU/mL). EBV, Epstein-Barr virus; IU, international units.

Table S5. Comparison of the EBV DNA levels attained using the NeuMoDx EBV Quant Assay and the artus EBV QS-RGQ Kit.

Sample No.	artus EBV QS-RGQ Kit		NeuMoDx EBV Quant Assay	
	Result	Concentration, IU/mL	Result	Concentration, IU/mL
Detected within the quantification limits of both assays, n = 16				
54	Detected	1,080.8	Detected	2,900
55	Detected	1,218.5	Detected	2,100
56	Detected	339.1	Detected	650
57	Detected	420.2	Detected	1,100
58	Detected	451.7	Detected	780
60	Detected	418.3	Detected	380
61	Detected	155.3	Detected	250
62	Detected	171.3	Detected	490
64	Detected	48.3	Detected	260
65	Detected	174.8	Detected	200
68	Detected	109.2	Detected	360
73	Detected	211.3	Detected	330
84	Detected	25.6	Detected	260
87	Detected	4,796.8	Detected	56,000
88	Detected	2,610.4	Detected	42,000
89	Detected	3,933.1	Detected	54,000
Detected above the artus LoD but detected below the NeuMoDx LoD, n = 2				
23	Detected	39.5	Detected	<200
81	Detected	33.2	Detected	<200
Detected below the artus LoD and detected below the NeuMoDx LoD, n = 6				
22	Detected	<22.29	Detected	<200
24	Detected	<22.29	Detected	<200
69	Detected	<22.29	Detected	<200
70	Detected	<22.29	Detected	<200
72	Detected	<22.29	Detected	<200
80	Detected	<22.29	Detected	<200
Not detected by artus but detected below the NeuMoDx LoD, n = 1				
77	Not detected	0.0	Detected	<200
Detected below the artus LoD but not detected by NeuMoDx, n = 12				
9	Detected	<22.29	Not detected	N/A
20	Detected	<22.29	Not detected	N/A
21	Detected	<22.29	Not detected	N/A
37	Detected	<22.29	Not detected	N/A
38	Detected	<22.29	Not detected	N/A
39	Detected	<22.29	Not detected	N/A
40	Detected	<22.29	Not detected	N/A
42	Detected	<22.29	Not detected	N/A
66	Detected	<22.29	Not detected	N/A
71	Detected	<22.29	Not detected	N/A
75	Detected	<22.29	Not detected	N/A
76	Detected	<22.29	Not detected	N/A
Not detected by artus and not detected by NeuMoDx, n = 38				
1	Not detected	N/A	Not detected	N/A
2	Not detected	N/A	Not detected	N/A
3	Not detected	N/A	Not detected	N/A
4	Not detected	N/A	Not detected	N/A

5	Not detected	N/A	Not detected	N/A
6	Not detected	N/A	Not detected	N/A
7	Not detected	N/A	Not detected	N/A
8	Not detected	N/A	Not detected	N/A
10	Not detected	N/A	Not detected	N/A
11	Not detected	N/A	Not detected	N/A
12	Not detected	N/A	Not detected	N/A
14	Not detected	N/A	Not detected	N/A
16	Not detected	N/A	Not detected	N/A
17	Not detected	N/A	Not detected	N/A
18	Not detected	N/A	Not detected	N/A
19	Not detected	N/A	Not detected	N/A
25	Not detected	N/A	Not detected	N/A
26	Not detected	N/A	Not detected	N/A
27	Not detected	N/A	Not detected	N/A
29	Not detected	N/A	Not detected	N/A
30	Not detected	N/A	Not detected	N/A
31	Not detected	N/A	Not detected	N/A
32	Not detected	N/A	Not detected	N/A
33	Not detected	N/A	Not detected	N/A
34	Not detected	N/A	Not detected	N/A
35	Not detected	N/A	Not detected	N/A
36	Not detected	N/A	Not detected	N/A
41	Not detected	N/A	Not detected	N/A
43	Not detected	N/A	Not detected	N/A
44	Not detected	N/A	Not detected	N/A
45	Not detected	N/A	Not detected	N/A
46	Not detected	N/A	Not detected	N/A
49	Not detected	N/A	Not detected	N/A
50	Not detected	N/A	Not detected	N/A
51	Not detected	N/A	Not detected	N/A
52	Not detected	N/A	Not detected	N/A
53	Not detected	N/A	Not detected	N/A
74	Not detected	N/A	Not detected	N/A

EBV, Epstein-Barr virus; IU, international units; LoD, limit of detection; N/A, not applicable.

References

10. NeuMoDx CMV Quant Test Strip: Instructions for Use. Available online: https://www.neumodx.com/wp-content/uploads/2020/09/IFU_201400_NeuMoDx%E2%84%A2-CMV-Quant-Test-Strip_40600165_D_US-Export.pdf (accessed on *31* May 2022).
11. NeuMoDx EBV Quant Test Strip: Instructions for Use. Available online: https://www.neumodx.com/wp-content/uploads/2021/07/IFU_201500_NeuMoDx%E2%84%A2-EBV-Quant-Test-Strip_40600294_E_US-Export.pdf (accessed on *31* May 2022).20. Artus® CMV QS-RGQ MDx Kit Instructions for Use (Handbook). Version 1. Available online: https://www.qiagen.com/us/products/diagnostics-and-clinical-research/transplant/artus-viral-load/artus-cmv-qs-rgq-mdx-kit-us/ (accessed on *31* May 2022).
23. Mourik, K.; Boers, S.A.; van Rijn, A.L.; Thijssen, J.C.P.; Doorn, R.; Svraka, S.; Bart, A.; Wessels, E.; Claas, E.C.J.; Nijhuis, R.H.T. Evaluation of the sample-to-result, random access NeuMoDx platform for viral load testing of Cytomegalovirus and Epstein Barr virus in clinical specimens. J. Clin. Virol. 2022, 149, 105122
26. Artus EBV PCR Kits CE. Available online: https://www.qiagen.com/gb/products/diagnostics-and-clinical-research/transplant/artus-viral-load/artus-ebv-pcr-kits-ce/ (accessed on *31* May 2022).
